# Evaluation of the Spermicidal and Contraceptive Activity of Platycodin D, a Saponin from *Platycodon grandiflorum*

**DOI:** 10.1371/journal.pone.0082068

**Published:** 2013-11-26

**Authors:** Zongliang Lu, Leiguang Wang, Rui Zhou, Yi Qiu, Liuna Yang, Chanyu Zhang, Min Cai, Mantian Mi, Hongxia Xu

**Affiliations:** 1 Department of Nutrition, Daping Hospital and Research Institute of Surgery, Third Military Medical University, Chongqing, PR China; 2 Shandong Provincial Family Planning Institute of Science and Technology, Jinan, PR China; 3 Research Center for Nutrition and Food Safety, Third Military Medical University, Chongqing, PR China; 4 The Second Affiliated Hospital of Chongqing Medical University, Chongqing, PR China; 5 Institute of Chongqing National Population and Family Planning Science, Chongqing, PR China; University of Miami School of Medicine, United States of America

## Abstract

**Background:**

The extract of *Platycodon grandiflorum* has been reported to have effective spermicidal activity. This study was designed to evaluate the spermicidal and contraceptive activity, as well as the safety, of Platycodin D (PD), a major saponin in *Platycodon grandiflorum*.

**Methods:**

Using the computer-aided sperm analysis (CASA) test criteria, the sperm-immobilizing activity of PD was studied using highly motile human sperm. The sperm viability was assessed by fluorescent staining using SYBR-14 (living sperm) and propidium iodide (dead sperm). The sperm membrane integrity was assessed by evaluating the hypo-osmotic swelling (HOS) and examinations by transmission electron microscopy (TEM) and scanning electron microscopy (SEM). The *in* *vivo* contraceptive efficacy was evaluated in rats using post-intrauterine PD application. The comet assay was employed to determine whether PD caused DNA damage in the sperm. Vaginal biopsies were also performed to determine whether the PD gel induced vaginal inflammation.

**Results:**

A dose-dependent effect of PD on the sperm motility and viability was observed. The maximum spermicidal effect was observed with a 0.25 mM concentration of PD. More than 70% of the PD-treated sperm lost their HOS responsiveness at a concentration of 0.20 mM PD, indicating that PD caused injury to the sperm plasma membrane. TEM and SEM revealed significant damage to both the head and tail membranes of the sperm. PD decreased the fertility to zero in rats, was non-DNA damaging and was not harmful to the vaginal tissue in the rats.

**Conclusion:**

PD has significant spermicidal activity that should be explored in further studies.

## Introduction

The explosive growth of the global population has been a major concern that poses a significant threat to the quality of life of people in various countries. There are more than 200 million pregnancies worldwide every year, and it has been estimated that more than half of them are unwanted [1]. Dual-contraceptive method use, defined as the simultaneous use of condoms and a non-barrier contraceptive [2], such as spermicide, as an important strategy for promoting reproductive health. Although many types of spermicidal contraceptives are currently available, they have side effects that decrease their applicability [3]. For example, it has been reported that the vaginal spermicidal agent, nonoxynol-9, causes genital ulceration, and thus increases the chance of HIV-1 infection upon repeated use [3,4]. 

Many medicinal plants have been reported to possess spermicidal activity [5-8]. The seed extracts of *Madhuca latifolia* were reported to have spermicidal activity at a 2% concentration [8], and Mi-saponin A is a potent spermicidal molecule derived from *Madhuca latifolia* that may be explored further as an effective component of a vaginal contraceptive [8]. Oleanolic acid 3-beta-D-glucuronide (OAG) was isolated from *Sesbania sesban* Merrill, a shrub belonging to the *Leguminosae* family, and was demonstrated to be useful as a spermicidal agent [9]. The extract from *Cestrum parqui* has also been demonstrated to have spermicidal activity, and the maximum spermicidal effect was observed with a 250 μg/mL concentration of the extract [10]. In addition, the essential oil of *Trachyspermum ammi* (L.) possesses appreciable spermicidal potential [11]. Qiu et al. reported that the crude extract of *Polygala tenuifolia* has rapid spermicidal activity *in vitro* [12]. Farnsworth and Waller [13] screened a large number of plants for spermicidal properties, and reported that the majority of plant-derived spermicides were triterpene saponins of several structural types. Pakrashi et al. have also reported that the majority of triterpenoid saponins of plant origin possess spermicidal properties [14]. Most of the plant spermicidal compounds act on the sperm surface and disrupt the sperm membrane [13]. 

*Platycodon grandiflorum* is a traditional Oriental medicine, which is widely used as an expectorant and as a remedy for respiratory disorders [15]. Platycodin D (PD) is the major anti-inflammatory constituent present in the root of *Platycodon grandiflorum* [16]. PD is a triterpene saponin that has recently been investigated as a candidate cancer chemotherapeutic agent [17]. Qiu et al. found that an extract from the *Platycodon grandiflorum* root and isolated PD both have rapid spermicidal effects [18,19]. The current investigation was undertaken to further evaluate the spermicidal and contraceptive efficiency of PD *in vitro* and *in vivo*, to explore its effects on the integrity of the sperm membrane, and to evaluate the safety of PD in terms of its ability to cause DNA and tissue damage. 

There have been reports demonstrating that the majority of plant-derived spermicides were triterpene saponins [13,14], so we compared the spermicidal effects of PD with those of several other triterpene saponins derived from different Chinese traditional medicines, including Onjisaponin B (OB), Hederasaponin B (HB) and Deapio playtcodin D (DPD). OB is a triterpene saponin from *Polygala tenuifolia*, the crude extracts of which have been reported to have rapid spermicidal activity *in vitro* [12]. HB is one of the triterpene saponins from *Acanthopanax senticosus* (AS), which was reported to improve the sperm motility of asthenospermia patients *in vitro* [20]. DPD is another triterpene saponin present in the root of *Platycodon grandiflorum* that has a similar structure to PD.

## Material and Methods

### Chemicals

**Figure 1:**
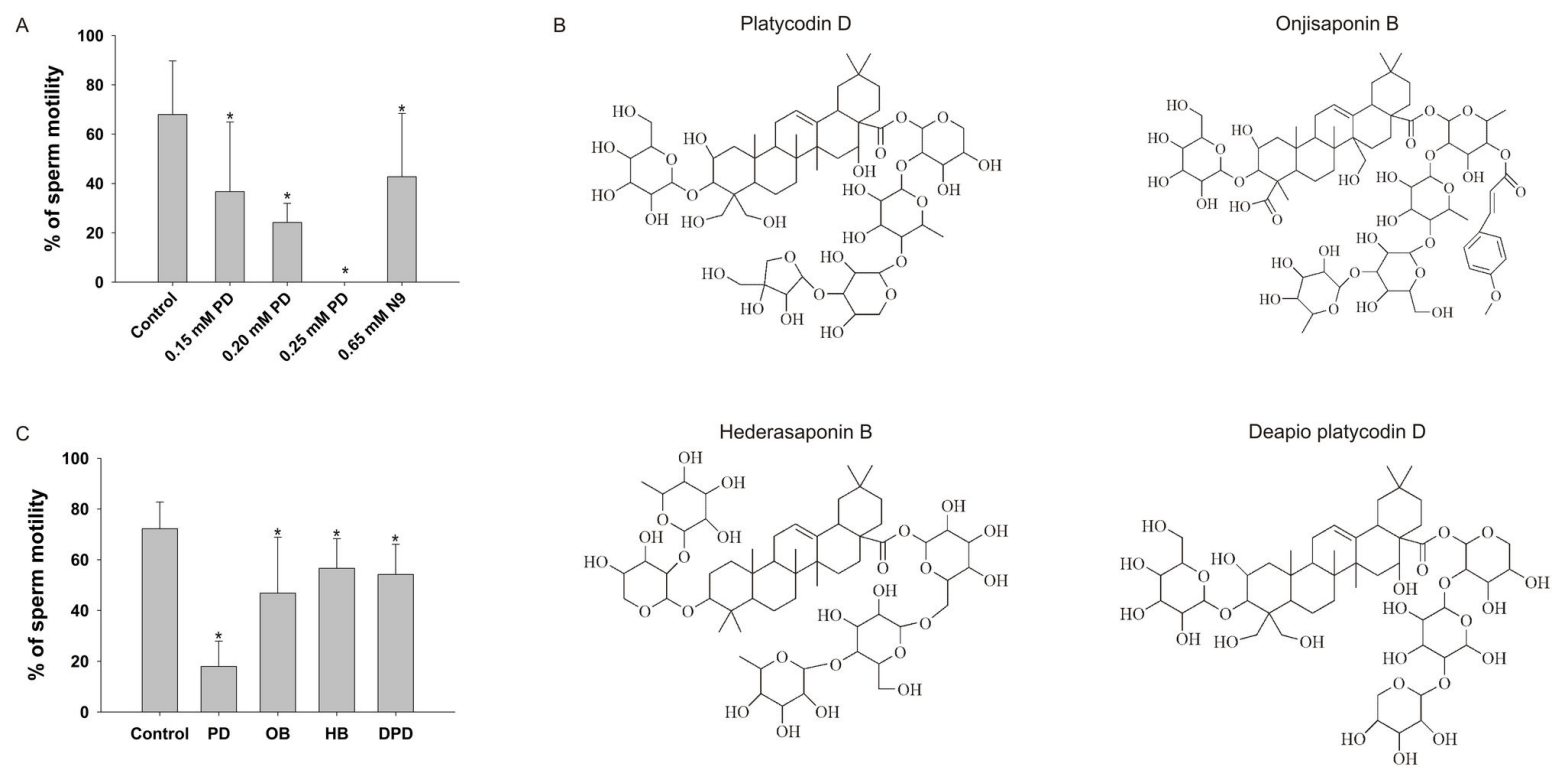
Effects of different concentrations of PD and different saponins on the sperm motility. (A) PD caused a dose-dependent immobilization compared to the control. A concentration of 0.65 mM of N-9 (= 20 mg/L) served as a positive control. The data represent the means ± SEM (n=12). (B) The structures of the four saponins, PD, Onjisaponin B (OB), Hederasaponin B (HB) or Deapio platycodin D (DPD) . (C) Sperm were treated with the same concentration (0.20 mM) of PD, OB, HB, and DPD. The 0.20 mM concentration of PD significantly decreased the sperm motility. The data represent the means ± SEM (n=8). *: Significantly different from the control, p<0.01.

PD, OB, HB and DPD were procured from Chengdu Must Bio-Technology Co., LTD (Chengdu, China). The structures of the four compounds are shown in Figure 1. The LIVE/DEAD sperm viability kit (cat. L-7011) was obtained from Invitrogen (Molecular Probes Inc., Eugene, OR, USA). The sperm Comet Assay Kit was from GenMed Scientifics (Boston, MA), and the HOS test kit was from Baso Diagnostics Inc. (Zhuhai, China). All other chemicals were purchased from Sigma (St. Louis, MO, USA). 

### Sperm collection and preparation

A total of 102 fresh human semen samples, collected by masturbation after a three-day period of sexual abstinence, were obtained from healthy males aged 23 to 41 years undergoing a routine semen analysis at the Institute of Chongqing National Population and Family Planning Science, Chongqing, China. The semen characteristics, volume, pH, viscosity, density, motility and morphology were determined based on the World Health Organization (WHO) guidelines [21]. The study protocols were approved by the Institutional Review Boards of the Third Military Medical University and the Institute of Chongqing National Population and Family Planning Science. All the participants provide their written informed consent to participate in this study. Samples with a normal morphology of more than 60% of the spermatozoa were used for this study. 

### Assessment of spermicidal activity

The spermicidal activities of different concentrations of PD were assessed by a computer-assisted sperm analysis system (CASA) [21]. The CASA system with phase contrast microscope optics is able to perform reliable assessments of sperm movement pattern characteristics in semen, and the percentages of motile and progressively motile spermatozoa can also be determined using the CASA technology. Different stock solutions of PD were prepared in 1% DMSO, and 1 μl of each stock solution was mixed with an aliquot of 99 μl of sperm, to provide a final concentration of 0.15 mM, 0.20 mM or 0.25 mM. A concentration of 0.65 mM nonoxynol-9(N-9) was used as a positive control. The specimens were examined by the CASA system after 20 s of treatment. The semen samples from 12 healthy fertile men were used for this experiment. The spermicidal activities of the three structurally-related saponins; OB, HB and DPD, were also assessed by a CASA system at the same concentration of 0.20 mM. Semen samples from eight additional healthy men were used for this experiment. 

### Hypo-osmotic swelling test

PD-treated and untreated spermatozoa were exposed to a HOS solution (75 mM fructose and 20 mM sodium citrate) for at least 30 min at 37°C to detect changes in the sperm membrane integrity [22]. The number of spermatozoa showing characteristic tail curling or swelling was counted under a phase contrast microscope (×100, Olympus BX51). Images were captured by an Olympus DP 71 camera system (Japan). Samples from six healthy fertile men were used for this experiment. 

### Sperm viability testing by fluorescent staining

Semen samples were obtained from five fertile men. Each sample was added to 2 ml of upstream fluid (0.47 mM sodium pyruvate, 12.86 mM sodium bicarbonate, 6.42 mM calcium chloride, 25 µg/ml gentamicin and 1.50% human albumin in F10 medium) and was incubated for two hours at 37°C. The sperm viability was determined using a LIVE/DEAD Sperm Viability Kit (L-7011, Molecular Probes, Eugene, OR) according to the manufacturer’s instructions, where live sperm fluoresced green (SYBR-14 dye) and dead sperm fluoresced red (propidium iodide, PI). PD-treated (0.10 mM or 0.20 mM), N-9-treated (0.20 mM) and untreated sperm samples were subjected to sperm viability assessment. The living and dead sperm count was determined in a sample of 100 spermatozoa for each group under a confocal microscope (Leica TCS SP5, Germany). 

### Ultrastructural study

For transmission electron microscopy (TEM), 2 ml of semen was mixed with 3 ml of F10 medium (HyClone, Logan, UT), and was incubated at 37°C for 1 hr. A total of 990 μl of the upper suspension was mixed with 10 μl of 15 mM PD (final concentration: 0.15 mM) or 10 μl of 0.1 M phosphate buffer (PBS, pH 7.2), followed by gentle centrifugation (2000 rpm, 8 min). The sediment was then washed with PBS. The sperm suspensions (both exposed to 0.15 mM PD and untreated) were fixed with 2% glutaraldehyde in 0.1 M PBS overnight, and post-fixation was done using 1% osmium tetroxide for observation under a transmission electron microscope (TECNAI 10, FEI company, Holland). 

Further, a scanning electron microscope (SEM) was used to observe the morphological changes and membrane degradation after treatment. The 0.15mM PD-treated and untreated samples used for the SEM analyses were prepared according to a previously reported protocol [23]. The spermatozoa were finally observed under a scanning electron microscope (s-3400N, Hitachi, Japan). 

### Comet assay

Human semen samples were obtained from five fertile men by masturbation. The semen samples were combined with 2 ml of upstream fluid and incubated for two hours at 37°C. Then, the upstream fluid was washed twice with PBS and resuspended in 500ul PBS. A total of 280 µl of the suspension were exposed to 20 μl of 2 mM PD, 20 μl of 10 mM Benzo [a]pyrene (Bap, as a positive control) or 20 ul PBS (as a negative control) for 30s. Then, 50 μl of the upstream fluid was resuspended with 50 μl of 0.7% low melting agarose at 37°C, placed on a slide (precoated with 0.8% normal melting agarose) and gently covered with a cover slip. The following procedures were performed according to a previously reported protocol [24]. Finally, cells stained with ethidium bromide were viewed at ×200 magnification with a fluorescent microscope. The length of the comet tails reflected the level of DNA damage [24]. 

### Animals

Adult Sprague-Dawley rats (female: 180-220 g, male: 200-250g) were acquired from the animal house of The Third Military Medical University. The animals were maintained under standard laboratory conditions (12:12, dark: light cycle) and were fed a standard diet. Water was supplied *ad libitum*. All animal experiments were performed following protocols approved by The Third Military Medical University’s Animal Care and Use Committee in compliance with the NIH Guide for the Care and Use of Laboratory Animals.

### Contraceptive efficacy

The contraceptive efficacy of PD was assayed in rats via the intrauterine administration of PD and subsequent evaluation of mating outcomes, according to a method modified from a report by Das, et al. [9]. In brief, sexually mature cyclic adult female Sprague-Dawley rats (n=16) were subjected to light ether anesthesia and one small mid-ventral abdominal incision was made, through which the uterine horns were gently pulled out. In the right horn, 100 μl of 3 mg/ml PD solution (in sterile physiological saline) was introduced through a tuberculin syringe fitted with a 24 Ga needle that penetrated through the cervical end of the right horn (treated horn). The contralateral uterine horn (left horn) received 100 μl of sterile physiological saline (control horn) using the same method. The incision was closed by sutures. The animals were maintained in individual cages. In the evening of the same day, the female rats were exposed to males of proven fertility at a 4:2 ratio. The presence of sperm in the vaginal lavage the next morning confirmed mating. Mated rats were sacrificed on Day 10 of gestation. The uterine horns were examined, and the number of implantation sites/fetuses was counted. 

### Effects of PD on the vaginal epithelium in rats

Twelve sexually mature cyclic adult female SD rats were divided into four groups, with three rats in each group. A concentration of 3 mg/g PD in gel (1.50% carbomer, 0.20% ethylparaben, 10.80% glycerine and 1.50% triethanolamine) was applied to the vaginas of one group of rats for 14 consecutive days. In control rats, a placebo gel was applied as a vehicle control group, and rats that were not treated with anything served as a natural control group. Another group of three rats was treated with the marketed formulation, but a lower concentration (8 mg/g), of N-9 gel. A total of 0.5 g of all the gels (PD-containing, vehicle-only and N-9) was taken up into a 1 mL syringe with a sterile slim flexible tube, and was administered into the vagina of each rat. Three animals each from the control and each of the treatment groups were necropsied on day 15 and the vaginas were excised. The vaginal sections were evaluated semiquantitatively. Four basic criteria were considered: epithelial ulceration, leukocyte infiltration, edema and vascular congestion. The magnitude of vaginal changes was rated according to a previously published technique [25]. All of the evaluations were performed by two separate pathologists in the Department of Pathology of Daping Hospital, The Third Military Medical University according to the Chinese National Standard [26]. A score of 0-16 (none to intense) was given for each of the criteria, and the results were compared with those of the natural control. A total score between 0 and 8 was considered to be acceptable [26]. 

## Results

### Sperm motility analysis

As determined using the CASA system, Platycodin D (PD) caused a dose-dependent, immediate immobilization of spermatozoa compared to the control (Figure 1A, n=12). N-9 served as a positive control. The minimum effective concentration (MEC) of PD that induced 100% immobilization of the sperm in 20 s was found to be 0.25 mM. We also compared the immobilizing effects of Onjisaponin B (OB), Hederasaponin B (HB), Deapio platycodin D (DPD) and PD at 0.20 mM (Figure 1B-C). All four compounds induced significant decreases in sperm motility compared to the control (n=8). The 0.20 mM concentration of PD decreased the sperm motility significantly more than 0.20 mM OB. 

### Sperm membrane integrity

**Figure 2:**
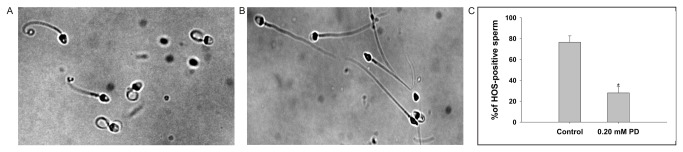
The HOS responsiveness of the sperm, as indicated by tail coiling. (A) The untreated control sperm and (B) sperm from the same man that had been treated with 0.20 mM PD were examined under a phase contrast microscope (injector: 20×). (C) The majority (76.5%) of the untreated control sperm were HOS-positive, whereas only 28.0% of the PD-treated sperm were HOS-positive. The data represent the means ± SEM (n=6). *:Significantly different from the control, *p*<0.05.

In the HOS experiments, the controls showed a high percentage (76.5±6.12%) of tail curling, while the tail curling of spermatozoa was significantly reduced in the samples treated with 0.20 mM PD (28.0±6.13%) (*p*<0.05) (Figure 2A-C). The loss of HOS responsiveness indicated compromised sperm membrane integrity post-PD treatment, suggesting an overall loss of sperm membrane physiology. 

### Sperm viability testing by fluorescent staining

**Figure 3:**
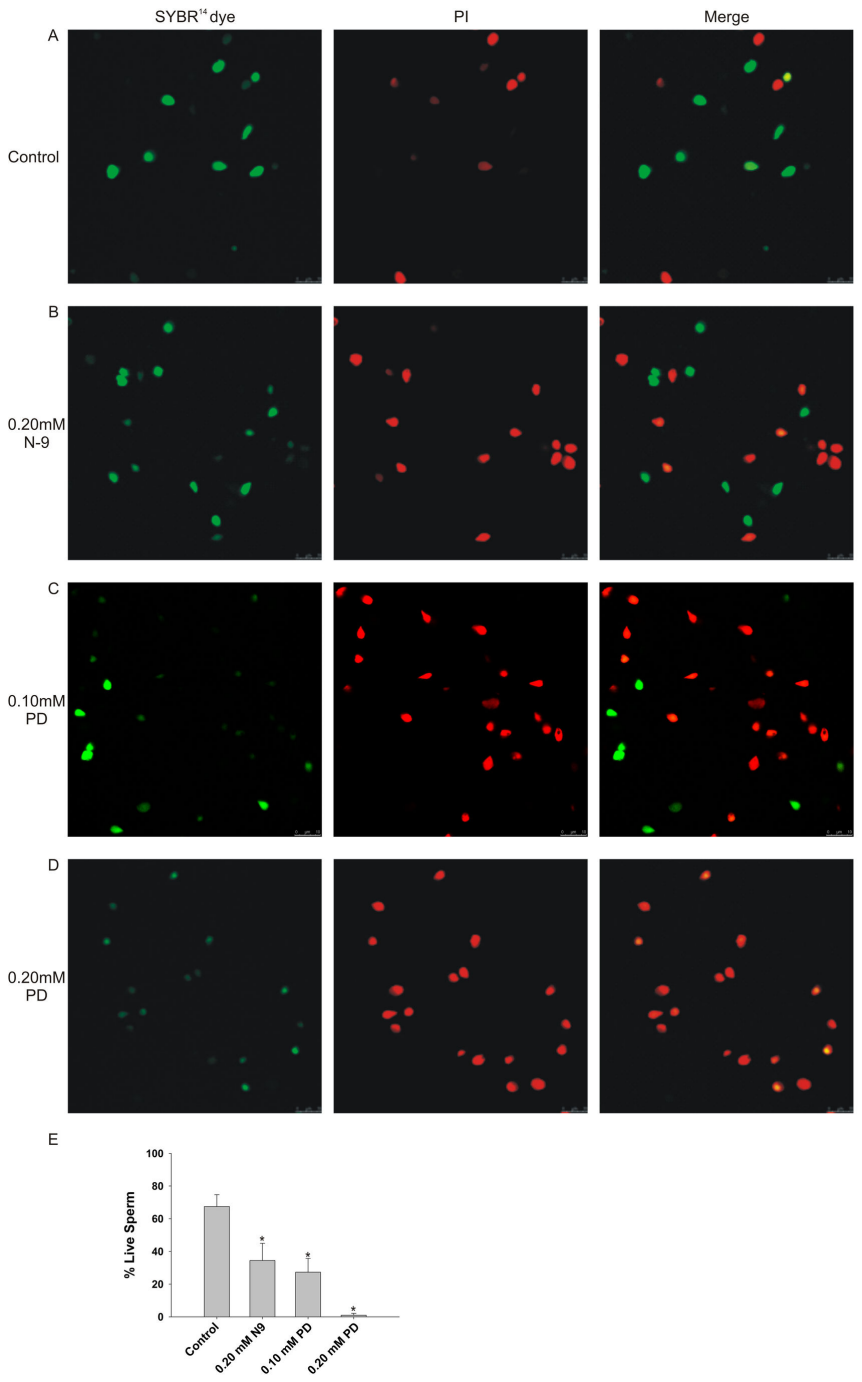
The sperm viability, as assessed by a fluorescent staining method. Live sperm cells fluoresced green (SYBR-14 dye), and dead sperm cells fluoresced red (PI) (1600×). (A) Control live sperm appeared green, confirming the utility of the assay. The spermatozoa treated with 0.20 mM N-9 (B) and 0.20 mM PD (C) both exhibited PI staining (red). (D) The sperm count showed that 0.20 mM PD killed almost all of the sperm, while 0.20 mM of N-9 only killed 65.5% of the spermatozoa. Each bar represents the mean ± SEM of six sets of observations; *: Significantly different from the control, p<0.05.

The LIVE/DEAD Sperm Viability Kit is a fluorescence-based assay used to analyze the viability and fertilization potential of sperm. Live spermatozoa with intact cell membranes fluoresce bright green (SYBR-14 dye), while cells with damaged cell membranes fluoresce red (PI). The sperm count showed that, in the controls (without PD or N-9 treatment), 67.5% of the sperm were viable (green stained). In the cells treated with the 0.20 mM concentration of PD, only 1% of the sperm were viable, while 34.5% of the sperm were still viable after 0.20 mM N-9 treatment (Figure 3). 

### Ultrastructural study

**Figure 4:**
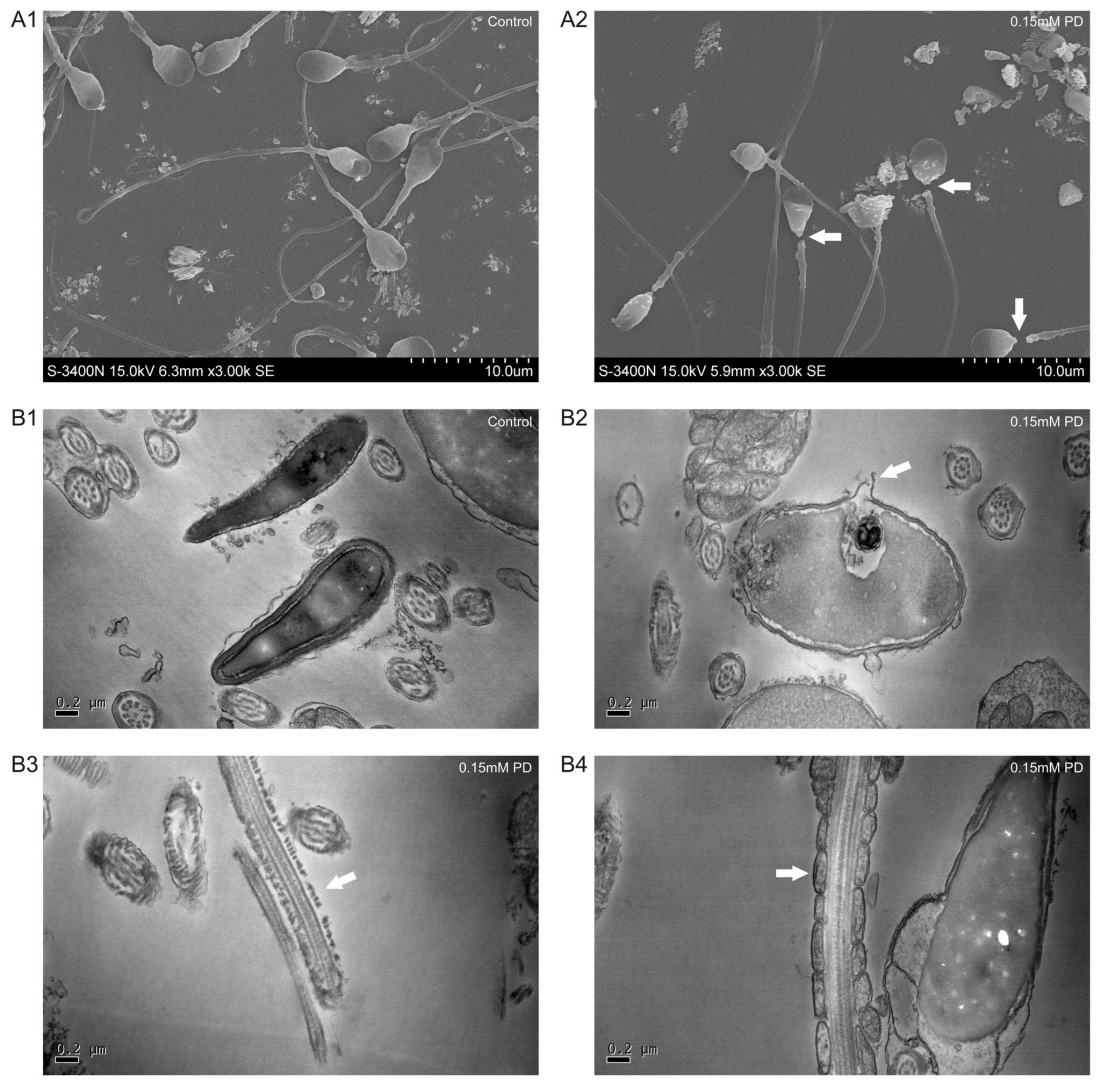
The microscopic ultrastructural changes in the sperm. Scanning electron microscopy (SEM) (A) and transmission electron microscopy (TEM) (B) images of untreated and PD-treated sperm. Normal sperm showing an intact membrane (A1), and 0.15 mM PD-treated sperm showing the detachment of heads (A2, arrows). Control sperm in the absence of PD showed intact membranes (B1), whereas the 0.15 mM PD-treated sperm showed membrane pore formation (B2, arrow), a missing tail membrane (B3, arrow) and swollen mitochondria (B4, arrow).

The SEM analysis demonstrated that the control sperm had a smooth surface and flat heads, as shown in Figure 4A. In contrast, cells treated with 0.15 mM of PD exhibited severe damage to the morphology of the cell membranes, showing vacuolation and the detachment of heads. The TEM analysis demonstrated that the control sperm samples exhibited intact plasma membranes without any appreciable morphological injury (Figure 4B), while the 0.15 mM PD-treated sperm showed severe damage due to membrane pore formation, a missing tail membrane and mitochondrial swelling of the tail.

### Contraceptive efficacy

**Figure 5:**
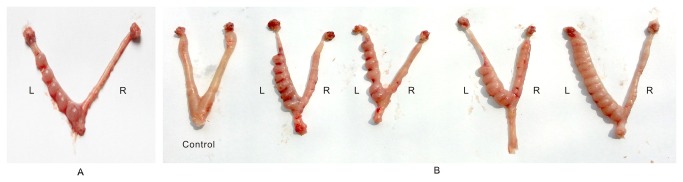
The contraceptive efficacy of PD in Sprague-Dawley rats. (A) A rat uterus with one physiological saline-treated (left) and one PD-treated (right) horn (B) The uterus from an untreated control rat (far left) and several additional Sprague-Dawley rat uteri, each with a physiological saline-treated horn (left) and a PD-treated horn (right).

All the rats mated successfully, and no changes in the mating efficiency were observed compared to normal rats. Not a single implantation site was observed in any of the rats in the PD-treated horns on Day 10 of gestation, while there were 5-12 implantation sites in the control horns. This observation (Figure 5) suggests that, even in the uterine milieu, PD could effectively block the sperm from reaching and/or fertilizing oocytes, while the fertilization and implantation were unhindered in the control horn. These results suggest that PD decreased the fertility to zero, without any systemic effects. 

### Effects of PD on the vaginal epithelium

The natural control group had a total score of 2.11±1.31 based on the pathological examination of the vaginal sections. The placebo gel led to a total score of 2.44±1.11. Treatment of the rats with the commercial N-9 gel resulted in a total score of 2.67±1.45. Similarly, the 3 mg/g PD gel treatment resulted in a total score of 3.78±1.31, and the differences between the four groups were not significant (p > 0.05), nor were there significant differences between the PD gel and placebo gel or between the N-9 gel and placebo gel (p > 0.05). For the scoring system [25], a score of 0-4 indicated mild vaginal irritation, indicating that the compounds had no significant effects on the vaginal epithelium at the concentrations used (Table 1). 

**Table 1:** The histological changes in rat vaginal tissue post-treatment.

**Parameters**	**Natural control**	**Placebo gel**	**PD gel (3 mg/g)**	**N-9 gel (8 mg/g)**
**Epithelial ulceration**	0.06±0.14	0.17±0.41	0.17±0.28	0.11±0.27
**Leukocyte infiltration**	0.50±0.62	0.61±0.85	1.44±0.72	0.83±0.94
**Vascular congestion**	1.17±0.75	1.33±0.42	1.50±0.46	1.56±0.89
**Edema**	0.39±0.25	0.33±0.30	0.67±0.47	0.17±0.18
**Total score**	2.11±1.31	2.44±1.11	3.78±1.31	2.67±1.45

All values are the means ±SD.

PD gel versus placebo gel, all five scores p > 0.05.

N-9 gel versus placebo gel, all five scores p > 0.05.

**Figure 6:**
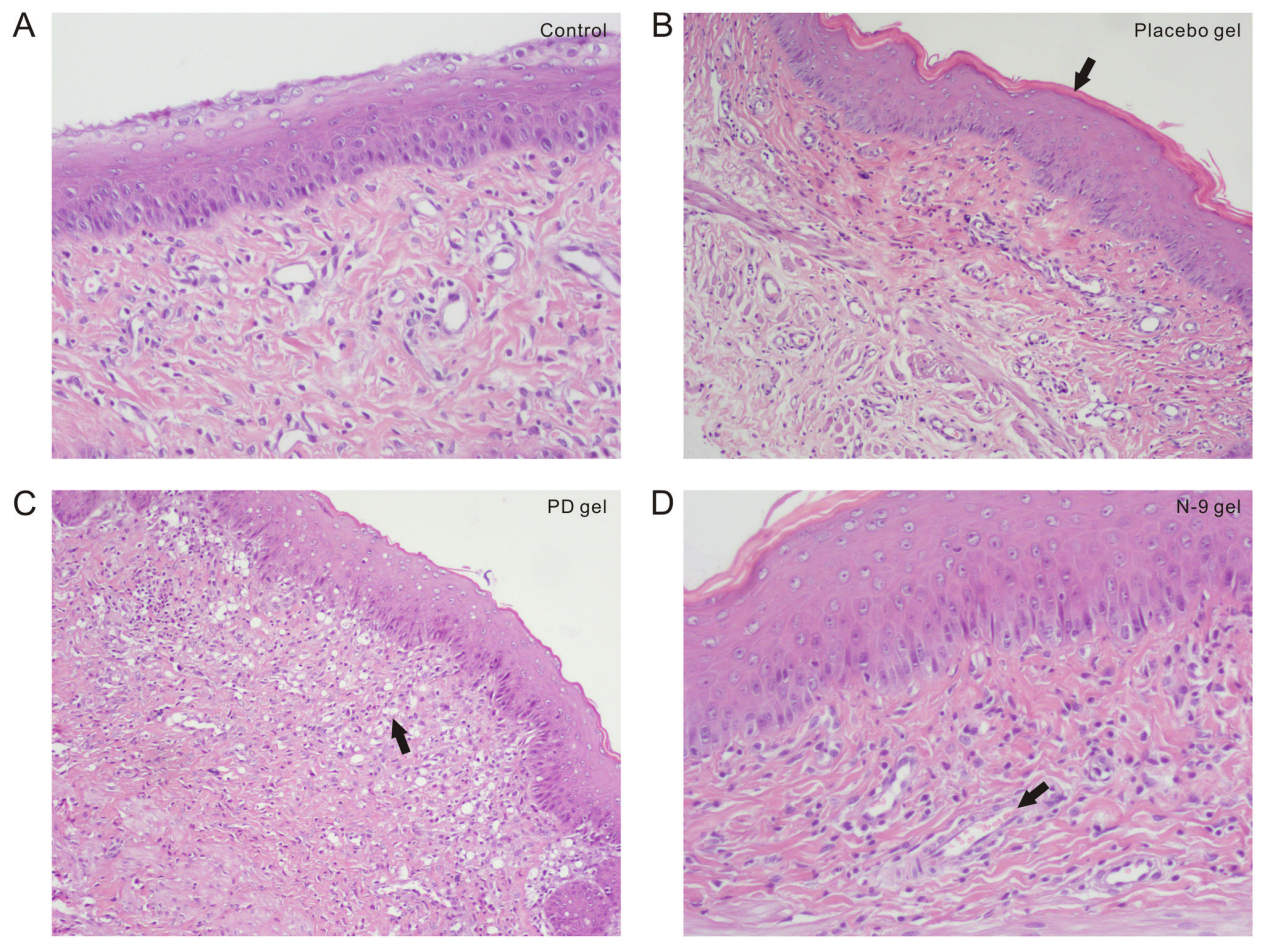
The histological changes in rat vaginal tissue following treatment with different gels. Photomicrographs showing the histological changes in rat vaginal tissue following (A) no treatment, or treatment with: (B) placebo gel, (C) 3 mg/g PD gel, which induced minor edematous thickening of the submucosal layer (arrow) or (D) 8 mg/g N-9 gel, which induced vascular congestion of the submucosal layer (arrow).

Intravaginal administration of a dose of PD that was five times the dose needed for contraception for 14 consecutive days induced a very minor inflammatory reaction in the vaginal epithelium, which included edematous thickening of the submucosal layer or infiltration of polymorphonuclear leukocytes into the mucosa. The N-9 gel induced vascular congestion of the submucosal layer at 1/5 the dose typically used for human contraception (Figure 6). However, the overall mean semiquantitative scores from the microscopic analysis showed that the changes did not exceed the definition for acceptability for any of the test groups [26].

### Mutagenicity study (comet assay)

**Figure 7:**
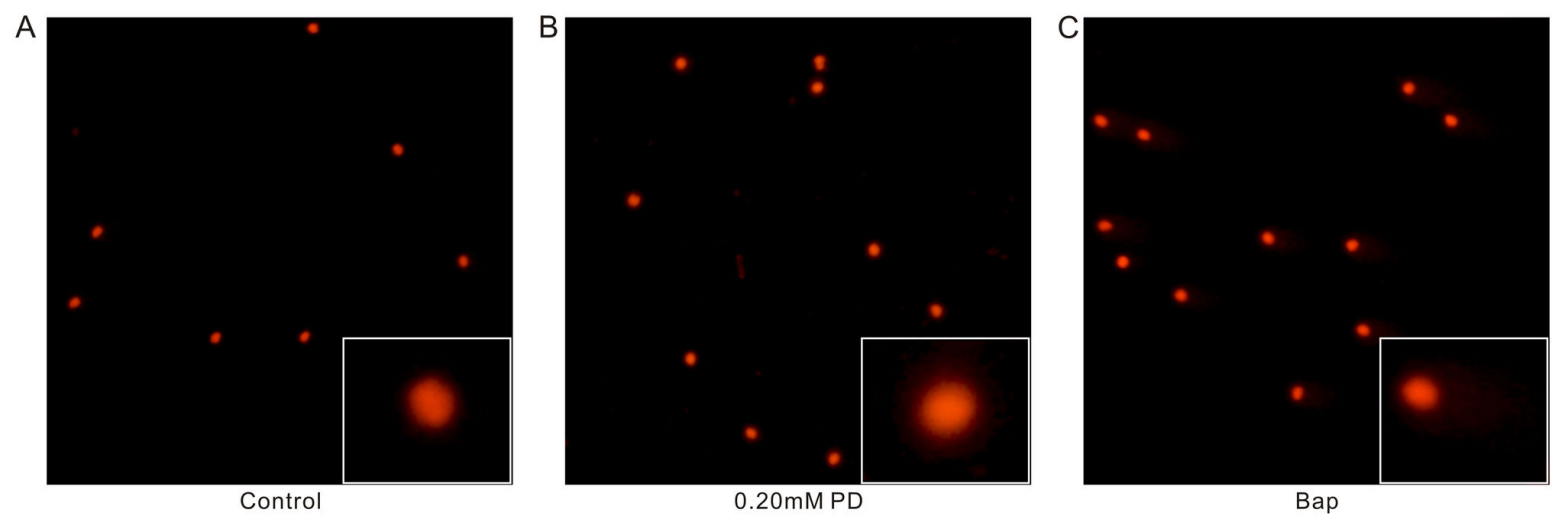
The DNA damage in the sperm, as assessed using the comet assay. (A) Untreated sperm and (B) 0.20 mM PD-treated sperm, which showed no DNA damage and (C) Bap-treated sperm (positive control), which exhibited significant DNA damage.

Using the comet assay, we examined the effects of 0.20 mM PD on the formation of DNA fragmentation in spermatozoa. Figures 7A and B show that the 0.20 mM concentration of PD did not induce any DNA damage compared to the untreated sperm cells. However, there was significant DNA damage in the Benzo[a]pyrene (Bap)-positive control (Figure 7C). This result indicates that the 0.20 mM dose of PD, the effective spermicidal concentration, induced no DNA damage in the sperm cells.

## Discussion

With an overall objective of developing a novel spermicidal agent that can serve as the active constituent of a vaginal contraceptive, the present investigation evaluated the spermicidal effects of PD by a series of *in vitro* and *in vivo* experiments. The results demonstrated that PD exerted a dose-dependent sperm-immobilizing effect and also decreased the viability of the sperm. The maximum spermicidal effect was observed with a 0.25 mM concentration of PD.

The plasma membrane plays a vital role in the process of sperm migration and fertilization. A number of spermicidal agents are known to execute their effects by inducing structural and/or functional modulation of the plasma membrane of spermatozoa. Most of the spermicidal compounds that have been identified from plants act on the sperm plasma membrane. For example, OAG, an active principle isolated from root extracts of *Sesbania sesban*, was reported to inhibit sperm motility and damage the sperm membrane integrity [9]. An extract from *Cestrum parqui* was revealed to induce significant damage to both the head and acrosomal membranes [10]. Chaudhury et al. reported that a concentration of 1 mg styrene maleic anhydride dissolved in 100 ml of DMSO caused significant damage to the acrosome and its contents, indicating a loss of the normal function of sperm [27]. We therefore examined whether the spermicidal effects of PD were mediated by modulation of the sperm membrane. Three commonly employed techniques were adopted; the hypo-osmotic swelling (HOS) test, an ultrastructural study and dual staining with SYBR-14 and PI.

The possibility that PD exerts its effects by inducing structural and/or functional modulation of the plasma membrane was supported by the differential reaction of the PD-exposed and unexposed sperm to a living cell nucleic acid stain, SYBR-14, and a membrane impermeable dye, PI. A morphologically intact membrane in live sperm offers selective permeability, and therefore blocks the entry of fluorescent dyes like PI. However, as the sperm die, they lose their ability to resist the influx of PI, which upon entering the sperm, either replaces or quenches the SYBR-14 staining and turns the sperm red. This forms the basis of the dual staining technique used to differentiate between live and dead sperm [28,29]. We observed that 32.5% of the total population of control spermatozoa showed PI staining after the dual fluorescence staining, while the remainder of the sperm appeared entirely green due to SYBR-14 staining. In contrast, exposure to 0.20 mM N-9 produced a significant decrease in the SYBR-14-stained green sperm population, with a corresponding increase in PI-stained red sperm (65.5%). However, at the 0.20 mM concentration of PD, almost all sperm were positively stained with PI, indicating that the compound killed almost 100% (99%) of the spermatozoa. Although this finding (shown in Figure 3) may appear to be in conflict with the findings shown in Figure 1A, where approximately 25% of the sperm still showed motility, the effects of many spermicides, including PD, are time-dependent [10,25]. Therefore, the apparent differences in the efficacy of PD were due to the differences in the experimental time course (20 seconds for the CASA studies and 30 minutes for the staining). 

The HOS test is an indicator of the structural and functional integrity of the sperm membrane [9,27]. When normal sperm are exposed to a hypo-osmotic environment, their intact plasma membrane permits the free passage of fluids into the cell so that it can reach osmotic equilibrium. As a result, the sperm volume increases, and the plasma membrane bulges. Since the plasma membrane around the sperm tail is more loosely attached than that around other parts, the tail responds to this increase in volume by coiling. This characteristic feature was exhibited in more than 75% of the sperm in the control group, while it was noted in only 25% of the sperm in the PD-treated group. This observation suggests that the functional integrity of the sperm membrane was lost following exposure to PD.

In the present study, the alteration of the sperm membrane integrity was confirmed by ultrastructural studies. Damage in the sperm head was clearly noted, suggesting a possible interaction of saponins with the plasma membrane sterols. Indeed, it has been reported that saponins form insoluble complexes with cholesterol and bile acids [30-34]. The hydrophobic portion of the saponin associates with the hydrophobic sterol nucleus in a stacked micelle aggregation. The interaction of saponins with cholesterol and other sterols plays a major role in many of the biological effects of saponins, particularly those involving membrane activities [35]. Consistent with these results, the electron microscopy findings also demonstrated that the effect of PD on the sperm membrane involved a loss of plasma membrane architecture, with the dissolution of the tail membrane. 

In the intravaginal toxicity studies, tissue irritation is usually evaluated by gross examination of the vaginal area, as well as by a complete histopathological evaluation [25,36]. In the present study, the 5-fold contraceptive dose (PD 3 mg/g gel) of the vaginal gel administered intravaginally for 14 consecutive days did not cause any gross lesions. Based on the histopathological examination following the treatment with PD gel, minor edematous thickening of the submucosal layer of the vaginal epithelium was observed, with a total score of 3.78±1.31, indicating marginal irritation, while treatment with the N-9-containing gel, at 1/5 the concentration used for human contraception, led to a total score of 2.67±1.45, consistent with a previous report suggesting that it causes vaginal irritation [35]. Since the scores were not significantly different between the PD and N-9 groups, the findings seem to indicate that the PD gel had a similar level of toxicity to the N-9 gel. However, the concentration of N-9 was actually 1/5 the clinically-used human concentration, while the concentration of PD was five times the concentration required for contraception in rats. In addition, we prepared the placebo gel, which was also used to deliver the PD in our lab, while the commercially-developed N-9 gel was obtained from the hospital pharmacy. Since the placebo gel itself resulted in a total score of 2.44±1.11, it is likely that the formulation of the gel was not optimal, and we will continue to explore new gel formulations to decrease the irritation caused by the gel itself. We believe that if PD were administered in an optimized gel, it would have a decreased effect on the vaginal epithelium, and would lead to a total score that is lower than that for the N-9 gel. 

The prospective contraceptive effect of PD was evaluated by intrauterine application in rats, and was encouraging, since the fertility on the treated side was reduced to zero. The rat is not a conventional model used to evaluate *in vivo* contraceptive efficacy, because semen is deposited in the cervix rather than in the vagina during fertilization [37]. However, the presence of the bicornuate uterus in the rat was useful, because one uterine horn served as a control for the other that was treated with the agent, while both horns were exposed to identical systemic milieu. PD will need to be tested in other animals following intravaginal application to confirm its contraceptive effects, but the clear differences between the uterine horns suggests that the compound effectively prevented conception, without eliciting systemic effects. 

Because there is contact between a spermicidal agent and human gametes, and because the components of the spermicide may be absorbed systemically, there may be concerns about the possible mutagenic effect of the compound [38]. Measurement of the DNA damage in spermatozoa is a useful tool to assess the toxicity and mutagenicity of compounds. The comet assay is a sensitive method used to measure DNA strand breaks [39,40], and we used this method to evaluate the possibility of mutation in sperm cells arising due to PD exposure. Sperm with high levels of DNA strand breaks would show an intense comet tail and an increased comet tail length due to increased DNA fragmentation [39,40]. Our data showed that the 0.20 mM concentration of PD did not induce any DNA damage compared to the untreated sperm cells. However, there was significant DNA damage in the Bap positive control, confirming the utility of the assay. These results indicated that the 0.20 mM concentration of PD, which is the effective spermicidal concentration, induced no DNA damage in sperm cells. Therefore, even if a sperm was to survive exposure to PD and fertilize an egg, it should not have acquired any mutations due to the PD. In addition, there would be no damage to the sperm if PD is absorbed systemically, nor should it lead to male infertility. 

In conclusion, this study has shown that PD possesses a remarkable spermicidal activity. Although the effective concentration is relatively high, it is similar to the range of other spermicidal saponins (8,9,11), PD is non-genotoxic, and the treatment with PD for 14 days did not cause any significant vaginal tissue damage, even at a concentration five-fold the dose needed for contraception. Further investigations, including *in vivo* studies of the spermicidal properties, are in progress. 
